# Ready-to-Use Nutraceutical Formulations from Edible and Waste Organs of Algerian Artichokes

**DOI:** 10.3390/foods11243955

**Published:** 2022-12-07

**Authors:** Nabila Brahmi-Chendouh, Simona Piccolella, Claudia Gravina, Marika Fiorentino, Marialuisa Formato, Naoual Kheyar, Severina Pacifico

**Affiliations:** 1 Laboratory of Biomathematics, Biochemistry, Biophysics and Scientometry, Faculty of Nature and Life Sciences, University of Bejaia, Bejaia 06000, Algeria; 2 Department of Environmental, Biological and Pharmaceutical Sciences and Technologies, University of Campania ‘Luigi Vanvitelli’, Via Vivaldi 43, 81100 Caserta, Italy; 3 Laboratory of Plant Biotechnology and Ethnobotany, Faculty of Nature and Life Sciences, University of Bejaia, Bejaia 06000, Algeria

**Keywords:** NaDES, *Cynara cardunculus* var. *scolymus*, polyphenols, simulated in vitro digestion, UHPLC-HRMS

## Abstract

Edible, plant-derived foodstuffs are recognized as precious sources of polyphenol compounds, whose consumption has proven to have multiple beneficial effects on human health. However, the awareness that cooking processes are able to induce quali-quantitatively changes in their native occurrence and that their bioavailability after food ingestion is poor led the research to move toward the preparation of nutraceutical supplements aimed at maximizing their content by effective extractive techniques and protecting them from degradation. The present work fits into this context, proposing a green, ready-to-use formulation of capitula, stems, and leaves of Algerian artichokes, in which natural deep eutectic solvents were exploited as extracting solvents but not removed at the end of the process. MTT test on the Caco-2 cell line highlighted that mitochondrial redox activity inhibition was absent below the 50 µg/mL tested dose. Simulated in vitro digestion was used as a predictive model for formulation bioaccessibility, where the joint approach with UHPLC-HRMS techniques allowed to define the release of each polyphenol from the investigated matrices. The capitula-based sample was the richest one in flavonoids, especially luteolin and apigenin glycosides, which survived in the intestinal digesta. On the contrary, simple phenols characterized the stem sample, whose release was mainly in the gastric chyme.

## 1. Introduction

The close connection between health and a correct lifestyle led the consumers to an increased awareness about several benefits provided by the daily intake of foods rich with bioactive and specialized metabolites within the diet. These compounds could be able to provide functional support to the human organism apart from nutrition, so much so that they could be claimed as nutraceutical substances. Among them, phenols and polyphenols have been recognized as main actors in exerting several biological activities (e.g., antioxidant, anti-inflammatory, anti-microbic, chemo-preventive, etc.) [1]. 

Polyphenols are ubiquitously biosynthesized in plants, and for this reason, they can be ingested through the diet from edible plants and derived foodstuffs. It has been widely demonstrated that agro-wastes and by-products also represent a rich but often undervalued source of these compounds [2]. Polyphenols from edible and not-edible parts of *Cynara cardunculus* var. *scolymus* (L.) Benth. (globe artichoke), previously simply known as *C. scolymus* L. (Asteraceae family), have been the focus of many phytochemical investigations [3,4,5,6] also related to the multiple beneficial effects for human health deriving from its consumption and reported in folk medicine, such as hepato-protective, choleretic, cardioprotective, and anti-cholinesterase [7]. Artichoke leaves have been recently included in a European Union herbal monograph highlighting the main chemical constituents claimed for their medicinal properties [8]. 

However, the use of artichoke for culinary purposes involves the cooking processes of edible parts, and it has been demonstrated that temperature is able to induce both qualitative and quantitative alterations in (poly)phenol composition [9], resulting in a reduced opportunity to exploit their health-related properties after ingestion.

In the last years, the development of dietary supplements containing nutraceutical polyphenols has aroused a huge interest in the scientific community. In this context, the enrichment of these bioactive ingredients has been achieved by means of efficient extractive methods from plant matrices using proper organic solvents (e.g., alcohols, hydro-alcoholic solutions, acetone). However, toxicity and environmental concerns dictated the need to move towards more sustainable extractants, among which natural deep eutectic solvents (NaDES) have gained particular attention. Their chemistry relies on the formation of hydrogen bonds between a donor and an acceptor that are natural substances, such as organic acids, sugars, quaternary ammonium salts, or betaine [10]. The NaDES extraction of plants materials, food samples, and food by-products has been the focus of an increasing number of published papers [11,12,13,14] (just to mention a few), proving to be responsible also for enhanced compound stability, bioactivity, and bioavailability [15], which make them suitable for applications in the nutraceutical sector besides pharmaceutical and cosmeceutical ones. To the best of our knowledge, the only investigation regarding deep eutectic solvents applied to *C. cardunculus* L. was aimed at improving the extraction yield of the sesquiterpene lactone cynaropricrin from the plant leaves [16].

The aim of the present work was to prepare ready-to-use food supplements containing bioactive compounds from three different organs (capitula, stems, and leaves) of an Algerian artichoke, taking advantage of NaDES extracting power towards polyphenols and avoiding their removal from the end product. To the best of our knowledge, this kind of approach has never been applied to globe artichoke. Capitula (or head) represent the immature inflorescence and is the common edible plant part, whereas stems and leaves are the main by-products deriving from industrial processing [17]. 

## 2. Materials and Methods

### 2.1. Plant Collection, NaDES Preparation, and Extraction

Plants of *Cynara cardunculus* var. *scolymus* (L.) Benth. were collected in April 2019 in the Béjaïa Province (Algeria) and identified by Dr. Adjir (Mentouri University, Constantine, Algeria). After harvesting, the plants were dissected into capitula, stems, and leaves; each organ was dried in the oven at 40 °C until a stable weight was achieved (after 7 days); and then, they were pulverized by a rotating knife homogenizer and stored at room temperature until use.

For the preparation of NaDES, choline hydrochloride (ChCl) and citric acid (CA) were anhydrified in the oven at 45 °C for one hour before use. Their mixture at a 1:1 molar ratio was heated under continuous stirring in a water bath at 80 °C for 30 min until a transparent solution was obtained. The solution was then diluted with distilled water (20%) and used as extractant of dried and pulverized artichoke organs (1:10, *w*:*w*). The extraction was accelerated by means of an ultrasonic bath (Ultrasonics^TM^ Bransonic^TM^ M3800-E, Danbury, CT, USA). Three cycles were performed, 30 min each, followed by dilution (1:1, *w*:*w*) and centrifugation at 4800 rpm for 20 min in an Avant^TM^ J-25 centrifuge (Beckman Coulter, Brea, CA, USA), equipped with a JA-14 rotor in order to remove the plant matrix. The free water was finally evaporated under vacuum until a constant weight was reached. Jelly-like formulations were obtained and stored at 4 °C until use.

### 2.2. Cell Culture and MTT Test

Human colorectal adenocarcinoma epithelial cell line (Caco-2) (ATCC^®^ HTB¬37TM, American Type Culture, Manassas, VA, USA) was cultured in Dulbecco’s Modified Eagle’s Medium (DMEM) supplemented with 10% fetal bovine serum, 50.0 U/mL of penicillin, and 100.0 μg/mL of streptomycin at 37 °C in a humidified atmosphere containing 5% CO_2_.

The cells were seeded in the medium in a 96-well plate at the density of 2.5 × 10^4^ cells/well. The day after, the cells were treated with the NaDES-artichoke samples at different concentrations in serum-free fresh medium (5, 10, 50, 100, and 200 µg/mL, final concentration levels) for 24 h. At the end of each incubation time, the MTT assay was performed as previously described [18]. Two independent experiments were carried out, performing in each six replicate measurements for three samples (*n* = 3) of the extract (in total, 6 × 3 measurements). Data were expressed as mean ± standard deviation (SD).

### 2.3. In Vitro Digestion Protocol

The simulation of in vitro digestion processes was conducted following the static method protocol proposed by the COST action INFOGEST network [19]. According to the recommendations, three saline solutions were prepared; designed to simulate salivary (SSF), gastric (SGF), and intestinal (SIF) fluids; and formed by the same saline constituents but mixed in different proportions and stored at −20 °C until use. The protocol consists of three phases, each one involving the addition of specific enzymes, as follows: Briefly, 5 g of NaDES-artichoke extracts was mixed with 3.5 mL of simulated salivary fluids (SSF) and 0.5 mL of α-amylase (prepared in SSF; final concentration 75 U/mL) previously heated at 37 °C, followed by 25 µL of a 0.3 M calcium chloride solution and 975 µL of distilled water. The pH was adjusted to 7 with NaOH 1 M, and the mixture thus formed was incubated at 37 °C for 2 min.

In a second experiment, carried out in parallel, in order to simulate the gastric phase, 7.5 mL of simulated gastric fluids (SGF) and 1.6 mL of a pepsin solution (prepared in SGF; final concentration 2000 U/mL) were added to 10 mL of oral bolus deriving from the simulated oral phase. Then, 5 µL of 0.3 M calcium chloride solution was further added and also 6 M HCl to bring the pH to 3, and finally, distilled water was added to obtain a final volume of 10 mL. The mixture was then incubated at 37 °C for 2 h in continuous stirring. The pH value was checked every 30 min in order to obtain the desired value if necessary.

In the third experiment, the digestion went further, and in order to simulate the intestinal phase, 11 mL of simulated intestinal fluids (SIF), 5 mL of pancreatin 800 U/mL (prepared in SIF; final concentration 100 U/mL), 2.5 mL of bile salts 160 mM (final concentration 10 mM), and 40 µL of CaCl_2_ 0.3 M were added to the gastric chyme. Subsequently, the pH was re-neutralized by NaOH 1 M, and finally, water was added to reach a 1:1 (v:v) ratio with gastric chyme. The mixture was then incubated at 37 °C for 2 h in continuous stirring. The pH value was checked every 30 min in order to obtain the desired value when necessary.

At the end of each stage of simulated in vitro digestion, the sample was immersed in liquid nitrogen to block any enzymatic activity and centrifuged at 4500 rpm for 10 min in an Avant^TM^ J-25 centrifuge (Beckman Coulter, Brea, CA, USA) equipped with a JA-14 rotor; the obtained supernatants underwent instrumental chemical analysis, as described below. 

### 2.4. UHPLC-HRMS Analyses of Parental Extracts and Digestate Therefrom

The chromatographic separation of samples (2 µL injection volume), carried out on a NEXERA UHPLC system (Shimadzu, Tokyo, Japan), was achieved on a Luna^®^ Omega C18 column (150 × 2.1 mm, 1.6 μm; Phenomenex), using 0.1% aqueous formic acid (solvent A) and acetonitrile (solvent B) as mobile phase. The linear gradient started at 2% B, was kept constant for 2 min, and then was led to 15% in 5 min, maintained for 5 min, and to 45% in the following 2 min. Then, after 0.5 min, the initial conditions were restored for re-equilibration. The flow rate was set at 0.5 mL/min. 

High-resolution mass spectrometry (HRMS) analyses were carried out by using the AB SCIEX TripleTOF^®^ 4600 spectrometer (AB Sciex, Concord, ON, Canada) equipped with a DuoSpray™ ion source operating in negative electrospray ion mode. The APCI probe was used for automated mass calibration in all scan functions using the Calibrant Delivery System (CDS). A non-targeted approach was developed combining TOF-MS and MS/MS with Information Dependent Acquisition (IDA), consisting of a full scan TOF survey (accumulation time 250 ms, 100–1500 Da) and eight IDA MS/MS scans (accumulation time 100 ms, 80–1300 Da). Other source and analyzer parameters were the following: curtain gas (CUR) 35 psi, nebulizer and heated gases (GS 1 and GS 2) 60 psi, ion spray voltage (ISVF) −4500 V, interface heater temperature (TEM) 500 °C, declustering potential (DP) 70 V, collision energy (CE) 35 V, and collision energy spread (CES) 25 V. For triterpenes, stronger DP and CE values were applied, which were 120 V and 100 (CES 25) V, respectively. The instrument was controlled by Analyst^®^ TF 1.7 software, while data processing was carried out using PeakView^®^ software version 2.2.

### 2.5. Antiradical Assays: DPPH and ABTS Tests

NaDES–artichoke samples and digestate therefrom were tested at 5, 10, 50, 100, and 200 µg/mL (final concentration levels) towards ABTS [2,20-azinobis-(3-ethylbenzothiazolin-6-sulfonic acid)] radical cation and 2,2-diphenyl-1-picrylhydrazyl (DPPH) radical.

ABTS radical cation was generated as previously reported [20]. The ABTS^•+^ solution was diluted with phosphate-buffered saline (PBS; pH 7.4) until an absorbance of 0.7 at 734 nm was reached. The extracts at different doses were directly dissolved in the ABTS^•+^ solution, and after 6 min, the absorbance was measured by a Victor3 spectrophotometer (Perkin Elmer/Wallac, Waltham, MA, USA) in reference to a blank, in which the samples were replaced with only NaDES.

DPPH^•^ scavenging capability was estimated as previously reported [20], and the absorption at 517 nm was measured on the Victor3 spectrophotometer in reference to a blank, in which the samples were replaced with only NaDES.

Trolox^®^ (2, 4, 8, 16, 32 μM) was used as the positive standard, and Trolox equivalent antioxidant capacity (TEAC) of samples was calculated based on both ABTS and DPPH tests. For each antiradical test, three replicate measurements for three samples (*n* = 3) of the extract (in total, 3 × 3 measurements) were performed. All data were expressed as mean ± standard deviation (SD).

## 3. Results and Discussion

NaDES were formed by CA and ChCl (1:1), as hydrogen bonds donor and acceptor, respectively. They are considered food-grade ingredients, and therefore, their removal is not necessary from extracts developed as food products for human consumption [21]. The preparation of potential ready-to-use formulations was achieved in four steps: (1) mixing of NaDES with the plant organs (capitula, stems and leaves); (2) sonication; (3) centrifugation; and (4) free water evaporation under vacuum. The final products obtained appeared as jelly-like formulations. The idea to consider them as ready-to-use food supplements with functional properties for human health cannot ignore their safety as well as their chemical composition (responsible for putative health-relative effects) and their fate during digestion, which could compromise their bioaccessibility.

### 3.1. MTT Test on Caco-2 Cell Line

In light of the above, the NaDES-based samples were at first tested on the human colorectal adenocarcinoma (Caco-2) cell line to obtain preliminary information about their capability to affect the cell mitochondrial redox activity (RAI), which represents the first sign of cell suffering due to cytotoxicity. Indeed, this cell line has been widely employed as a model of the intestinal epithelial barrier, especially in studies dealing with the absorption of dietary components [22]. 

**Figure 1:**
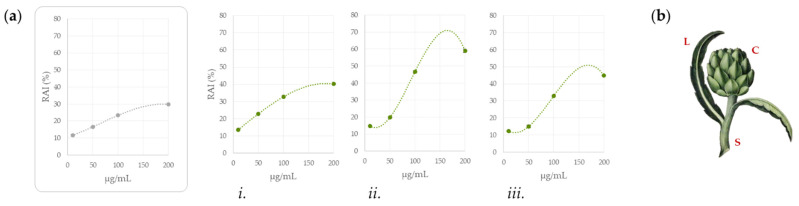
(**a**) Redox activity inhibition (RAI, %) by MTT test of carried out on Caco-2 cells after 24 h of exposure to NaDES-based food supplements from artichoke: (i) capitula, (ii) stems, and (iii) leaves (in the grey box the contribution of only NaDES to RAI% is reported). (**b**) Picture representation of artichoke organs (L, leaves; C, capitula; S, stems).

Caco-2 cells were exposed to increasing dose levels (5–200 µg/mL) of NaDES-based samples prepared from the three different artichoke organs (capitula, stems, and leaves). After 24 h MTT test was performed, and the results are reported in Figure 1. The cell mitochondrial redox activity appeared mildly compromised in a dose-dependent manner for all the extracts, and below the 50 µg/mL tested concentration, its inhibition could even be considered absent. Among the organs, the stem-based sample appeared more effective in the inhibition, whereas the leaves and capitula showed almost superimposable dose-response curves, with the latter being responsible for a maximum RAI, equal to 40.2 ± 0.06 %, only at the highest tested dose. The comparison with data obtained for NaDES alone, which were used as control, highlighted their poor contribution to the exerted effects. The preliminary results paved the way for considering the artichoke-based supplements as putative ready-to-use formulations once their chemical composition, which is responsible for the observed activity, are unraveled. 

### 3.2. UHPLC-HRMS Profiling of Artichoke-Based Food Supplements

All the samples underwent UHPLC-HRMS and MS/MS analyses to disclose their bioactive constituents. Apart from signals ascribed to citrate in the first 2 min (herein not discussed), 38 specialized metabolites were tentatively identified as 15 simple phenols, 21 polyphenols, and 2 triterpenes, all differently distributed. In Table 1, chromatography and mass spectrometry data that are useful for their tentative identification are summarized, whereas details are reported below for the compounds grouped in sub-classes.

#### 3.2.1. Simple Phenols

Simple phenols were depsides of hydroxycinnamic acids and quinic or methylquinic acids, differing in the structure and number of hydroxycinnamoyl residues and also in esterification position. TOF-MS/MS spectra of compounds **1** and **2** were almost superimposable, sharing the only fragment ion at *m*/*z* 191.0563 (59). Their elution order, together with previous data, allowed us to tentatively identify them as 1-*O* and 5-*O*-caffeoylquinic acid [23]. Two constitutional isomers of *p*-coumaroylquinic acid at *m*/*z* 337.0929 (**3** and **4**, C_16_H_18_O_8_) were also detected, whose MS/MS spectra evoke those of caffeoyl derivatives, as the main neutral loss gave rise to quinate ion at *m*/*z* 191.0560, which underwent dehydration, giving the ion at *m*/*z* 173.0441 (Figure S1). Compounds **5**, **20**–**22**, **26**, **28**, and **33** were recognized as dicaffeoylquinic acid (diCQA) regioisomers and putatively distinguished based on their mass fragmentation patterns and comparison with previously reported MS data [24,25]. Indeed, the absence of dehydrated quinate (at *m*/*z* 173.04) in MS/MS spectra suggested esterification occurring at C-1 and/or C-3 and/or C-5 positions. Thus, isomers **5**, **21**, and **22** were tentatively identified as 1,3-diCQA (also known as cynarin), 3,5-diCQA, and 1,5-diCQA, respectively. Except for metabolite **26**, which seemed to be a geometric isomer, the other ones were 4-*O*-acyl derivatives, namely 1,4-, 3,4-, and 4,5-diCQA (**20**, **28**, **33**).

**Figure 2:**
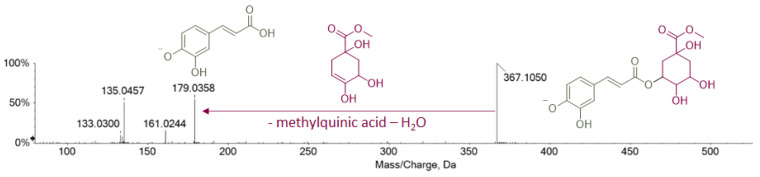
TOF-MS/MS spectrum of compound **6** with highlighted pivotal structures.

Compounds **6** and **10** showed the molecular formula C_17_H_20_O_9_ with an RDB value of 8. Although from the TOF-MS spectra, they could resemble feruloylquinic acid isomers, their TOF-MS/MS allowed us to discard this hypothesis. In fact, as depicted in Figure 2, fragment ions deriving from the collision-induced dissociation of the precursor ion were in accordance with the presence of a caffeoyl moiety. Thus, taking into consideration the neutral loss of 188 Da ascribable to dehydrated methylquinic acid, these metabolites were tentatively identified as caffeoyl methylquinate isomers. 

Analogously, compounds **32** and **35** were putatively identified as dicaffeoyl methylquinate isomers. It is worthy of note that when the carboxylic group of quinic acid was not free, the corresponding deprotonated fragment ion (methylquinate, at *m*/*z* 205.0718, calc. mass) was never generated. This observation could be pivotal for the straightforward discrimination of this molecular skeleton.

#### 3.2.2. Polyphenols

All polyphenol compounds were flavonoids, whose putative identity is discussed below, grouping them into three subclasses referring to the aglycone core. The detected 14 flavones were luteolin and apigenin derivatives, differently glycosylated. Compound **7** was identified as a hexosyl-hexuronyl derivative of apigenin. In fact, its TOF-MS/MS spectrum showed sequential neutral losses of 162.05 and 176.03 Da, leading to the disclosure of the deprotonated aglycone ion (at *m*/*z* 269.04) and the corresponding radical (at *m*/*z* 268.0378) (Table 1), whose intensity ratio allowed us to hypothesize a 7-*O*-hexuronidation and a 4′-*O*-hexosylation (Figure S2). On the contrary, TOF-MS/MS of metabolite **23** showed a reversed ratio between the two aglycone ions, suggesting an apigenin 7-*O*-hexosyl derivative (C_21_H_20_O_10_). Moreover, apigenin 7-*O*-hexuronide (**24**) and 7-*O*-rutinoside (**25**) were putatively identified, the latter being characterized by a neutral loss of 308 (162 + 146) Da (Figure S2) [26]. Finally, compound **36** could be in accordance with apigenin methylhexuronide. Indeed, the absence of a fragment ion at *m*/*z* 299, allowing us to exclude the possibility of a methylluteolin as aglycone, seemed to confirm the structural hypothesis. Instead, this latter fragment was observed in the TOF-MS/MS spectrum of compound **31**, putatively identified as methylluteolin (e.g., chrysoeriol; [27]) hexuronide, and of its *N*-derivative **15**, whose complete identity still remains unknown. 

A similar glycosylation pattern was observed for luteolin derivatives **8** (4′-*O*-hexosyl-7-*O*-hexuronidyl), **17** (7-*O*-hexuronidyl), **18** (4′-*O*-hexosyl), **27** and **29** (7-*O*-hexosyl; e.g., cynaroside), and **19** (7-*O*-rutinosyl; e.g., scolymoside). Moreover, a disaccharide formed by a pentose and a hexose (294 Da) characterized luteolin 7-glycoside **16**, likely bearing an *O*-arabinosyl-glucose moiety and previously characterized in *Carduus argyroa* and *Carduus nutans* subsp. *macrocephalus* samples [28].

**Figure 3:**
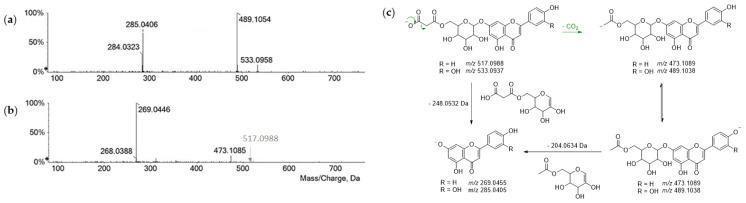
TOF-MS/MS spectra of compounds (**a**) **30** and (**b**) **34**. (**c**) Putative fragmentation pathway (theoretical *m*/*z* values are reported below each structure).

Flavones also occurred as malonylhexosides. They could be distinguished from the detection of a neutral loss of 248.05 Da (malonylhexose—H_2_O) from the deprotonated molecular ion [29] besides decarboxylation (Figure 3). 

Flavanone and flavonol glycosides were also detected. In particular, based on the occurrence of characteristic fragment ions at *m*/*z* 151, 135, and 107 (Figure S3), the aglycone of compounds **9**, **11**, and **12** was recognized as eriodictyol [30]. The saccharidic moiety, derived from neutral losses from deprotonated molecular ions, corresponded to hexose, hexuronic acid, and rutinose, respectively. Finally, TOF-MS/MS of compound **14** was in accordance with a quercetin 4′-*O*-hexoside due to the intensity ratio of deprotonated and radical aglycone ions (at *m*/*z* 301.035 and 300.027; [31]), whereas compound **13** was identified as the flavonol 3-*O*-hexuronide derivative [32].

#### 3.2.3. Triterpenes

**Figure 4:**
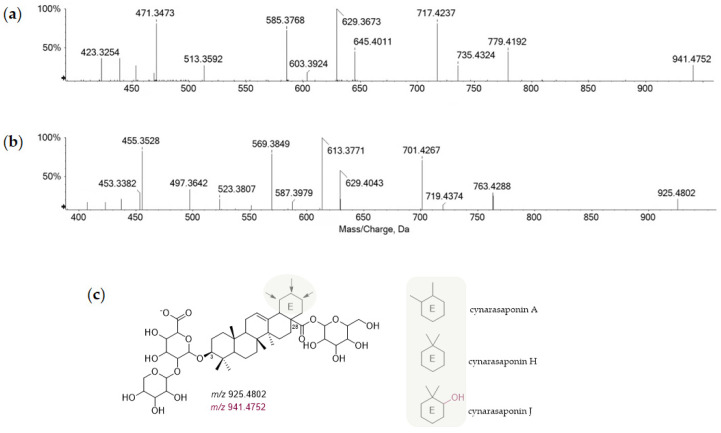
TOF-MS/MS spectra of compounds (**a**) **37** and (**b**) **38.** (**c**) Putative identification based on ring E structural variability.

The presence of two triterpene saponins was highlighted only in the artichoke capitula sample (**37** and **38**, Table 1). Both molecules appeared deprotonated in the TOF-MS spectrum, suggesting the presence of an oxidized sugar on the molecular skeleton, in accordance with previously published guidelines by HR-MS/MS tools [33]. To obtain additional information useful for their identification, stronger TOF-MS/MS declustering and collision energy potentials were applied. Both compounds shared the same saccharidic moieties, represented by a hexose bound to the C-28 carboxylic group and a (pentosyl)hexuronidyl residue linked at the C-3 position. The aglycone ion of saponin **37** showed a further hydroxyl group, likely at the C-21 position, enhancing its polarity and thus decreasing its retention time. In Figure 4, the HR-MS/MS spectra are reported, with tentative structures based on the variability of ring E, whereas in Figure S4, their hypothesized fragmentation pathways were depicted taking into account cynarasaponin H and J.

#### 3.2.4. Occurrence and Relative Content of Detected Bioactive Metabolites

**Figure 5:**
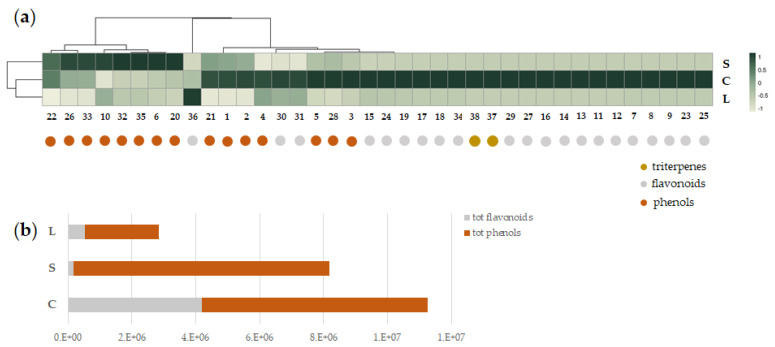
(**a**) Heatmap of the tentatively identified compounds in the ready-to-use food supplements made up of artichoke stems (S), capitula (C), and leaves (L). (**b**) Total relative content of each supplement in simple phenols and flavonoids based on peak areas.

Apart from cynarasaponins, which peculiarly characterized the capitula sample (Table 1, Figure 5a), simple phenols and flavonoids were differently distributed among the obtained products both qualitatively and quantitatively. As a general observation, the relative amount of bioactive (poly)phenols decreased in the order C > S > L (Figure 5b).

The capitula-based formulation was the richest one in flavonoids, with the only exception of apigenin methylhexuronide (**36**), whose content was about 2.5-fold higher in the leaf sample. Flavone glycosides were the most representative, followed by flavanone and flavonol derivatives. The latter ones (**13**,**14**) were extracted only from artichoke heads, together with eriodictyol hexuronide (**11**) and rutinoside (**12**). 

On the contrary, most of the phenols accounted for stems, in particular, caffeoyl- and dicaffeoyl-methylquinic acids and dicaffeoyl-quinic acids. 

### 3.3. In Vitro Digestion of Artichoke-Based Food Supplements 

The bioaccessibility of phenols and polyphenols within the artichoke-based food supplements was evaluated by means of a joint approach between in vitro digestion and UHPLC-HRMS techniques. For this purpose, the protocol proposed by the COST action INFOGEST network [19] was applied, the relative quantitation of each compound was performed at the end of each phase (oral, gastric, and intestinal), and their bioaccessibility was calculated in terms of release index from the matrix based on peak areas (Table S1). The amount of phenols and polyphenols released from the food matrix during the simulated digestion process has been commonly measured determined by means of colorimetric assays (e.g., Folin–Ciocalteu method for total phenol content—TPC) and also indirectly measured in terms of antioxidant activity [34]. However, it should be noted that these tests are not properly specific for phenolic compounds, as they are based on the chemical reactivity of different compounds also other than phenols [35]. Just to give an idea, suffice it to say that the Folin–Ciocalteu assay was first developed to detect the aromatic amino acid tryptophan, which, although it is not a non-phenolic compound, is able to reduce the reagent and increase the measured absorbance value. Thus, the mentioned method could be useful to compare samples in a preliminary screening, but in the case of in vitro assessment of food bioaccessibility, where some enzymes are added to the simulated fluids, it could give rise to overestimated results. On the contrary, chromatographic analyses coupled with MS detection are able to show a real picture of the digesta chemical composition without interferences. 

**Figure 6:**
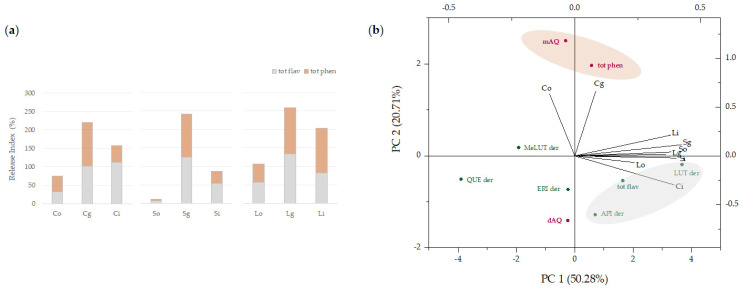
(**a**) Release index from the matrix (%) of total phenols and flavonoids relatively quantified by UHPLC-HRMS analysis. (**b**) Principal component analysis (PCA) of detected compounds, grouped in classes and sub-classes, referred to in vitro digestion phases. Co, capitula oral phase; Cg, capitula gastric phase; Ci, capitula intestinal phase; So, stems oral phase; Sg, stems gastric phase; Si, stems intestinal phase; Lo, leaves oral phase; Lg, leaves gastric phase; Li, leaves intestinal phase.

In Figure 6a, the release index from the matrices under study is reported, grouping the compounds into phenol and flavonoid classes. Moreover, the results were also analyzed by principal component analysis (PCA), further dividing the two main classes into sub-classes based on the number of acyl residues linked to quinic acid (mAQ, monoacyl quinic acid; dAQ, diacylquinic acid) or on the aglycone skeleton (LUT, luteolin; API, apigenin; ERI, eriodictyol; QUE, quercetin; MeLUT, methylluteolin) (Figure 6b). 

It appears clear that phenols from capitula, stem, and leaf NaDES-based food formulations are mainly released in the gastric chyme, whereas, among flavonoids, luteolin and apigenin derivatives from capitula were positively correlated to the final digesta (Ci). The data obtained are in accordance with previous results that emphasized the increase of artichoke monocaffeoylquinic acids in the gastric environment, whereas the transition to the intestinal conditions causes a significant increase in the total polyphenol recovery [36]. Indeed, the changes in the chemical composition of ingested foods are closely connected with the processes that take place at the digestive level, which modify the structures of the bioactive compounds and at the same time maintain or modify their biological activity [37]. In fact, bioactivity as well as cell absorption are closely related to their chemical structure. The bioaccessibility of (poly)phenols is influenced by several factors, such as chemical structure, the food matrix containing them, interaction with other components, or the presence of suppressors or cofactors [38]. Therefore, it is reasonable to assume that the peculiar composition of each organ as a whole is able to influence the bioaccessibility of individual compounds. As an example, regarding capitula samples, among apigenin derivatives, demethylation reactions of the methylhexuronidyl moiety of compound **36** could explain the enhanced relative content of compound **24** (hexuronide derivative) at the end of the digestion. Moreover, the massive release in the simulated gastric environment of apigenin hexosyl-hexuronide (**7**) and its successive degradation could be in line with the observed enhanced availability of compound **23** and, again, of metabolite **24**, which maintain only one of the glycosidic moieties (hexose or hexuronic acid, respectively). Quercetin glycosides were the only flavonols detected in the capitula-based formulation, whose integrity was massively affected in the gastric phase, so much so that they were almost absent in the final digestion step (Table S1).

### 3.4. DPPH and ABTS^+^ Radical Scavenging Capacity

**Figure 7:**
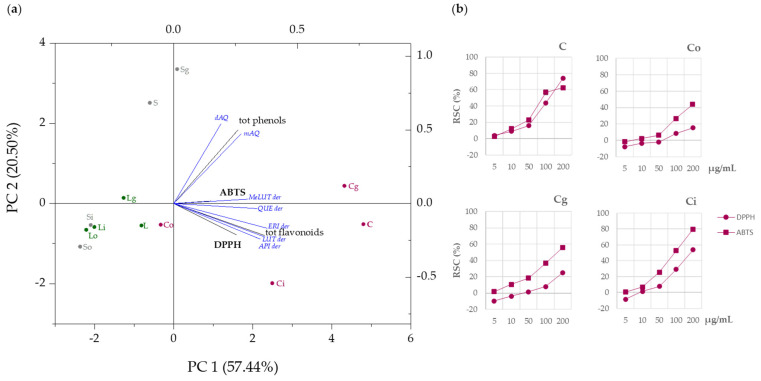
(**a**) Principal component analysis (PCA) of ready-to-use formulations before and after simulated digestion protocol, referred to DPPH and ABTS tests and the main compound sub-classes detected. (**b**) Radical scavenging capacity (RSC %) of capitula samples against DPPH and ABTS^+^ radicals (the corresponding TEAC values are reported in Table S3). C, not digested capitula; Co, capitula oral phase; Cg, capitula gastric phase; Ci, capitula intestinal phase; S, not digested stems; So, stems oral phase; Sg, stems gastric phase; Si, stems intestinal phase; L, not digested leaves; Lo, leaves oral phase; Lg, leaves gastric phase; Li, leaves intestinal phase.

The radical scavenging capacity of the undigested ready-to-use food supplements was evaluated by DPPH and ABTS^+^ radical tests and compared to the digested samples in the three stages. NaDES alone were tested at the same final doses to exclude their influence in the tests. The dose-dependent capability was verified in all cases, with capitula formulations before simulated digestion exerting the highest radical scavenging capacity of both radical probes, followed by leaf- and stem-based samples, respectively (Table S2). Following the three phases of simulated digestion, different responses were recorded, which likely remarked the peculiar quali-quantitative composition in terms of compounds’ release percentage. The PCA biplot reported in Figure 7a, able to describe the 77.94% of data variability, underlines the correlation between the investigated samples and the observed RSCs also in relation to the occurrence of different (poly)phenol sub-classes. Capitula gastric and intestinal digesta preserved their efficacy, which likely positively correlated to total flavonoid content, estimated in particular as the sum of apigenin and luteolin glycosides detected after digestion (Figure 7) beyond 1,3-dicaffeoylquinic acid (cynarin). On the contrary, stem-based samples, which mostly accounted for simple phenols, were located on the left-hand side of the graph, suggesting an inverse correlation to both radical tests. In particular, the lowest efficacy against the two probes, shown by So and Si samples, could be explained considering the overall very poor metabolite release compared to Sg. 

The antiradical behavior of (poly)phenol-enriched samples was in accordance with Jiménez-Moreno et al. [39], who underlined a high and significant correlation between the phenolic composition of artichoke waste extracts evaluated by HPLC and the antioxidant capacity exerted against radical probes. In line with previous research, the DPPH and ABTS methods were herein employed to further corroborate chemical composition data before and after simulated digestion, as they are able to provide accurate results strongly related to the sample chemical features. In fact, these tests are based on a mixed mode of action of a sample in transferring a single electron (SET) and/or a hydrogen atom (HAT) to the radical probe, resulting in its neutralization [22,40]. Thus, only compounds that possess active groups (e.g., catechol moieties in simple phenols and polyphenols) are able to react.

Indeed, the antioxidant properties of artichokes have been extensively correlated to the occurrence of flavonoids and chlorogenic acids [41,42,43,44]. In particular, the role of luteolin-7-*O*-glucoside (cynaroside) in reducing oxidative stress and inflammation was demonstrated both in vitro and in vivo [45,46,47]. 

## 4. Conclusions

The work reported herein showed that both edible and waste organs from artichokes could be exploited in the development of nutraceutical formulations. The use of NaDES as green extractive solvents promoted their enrichment in bioactive phenols and polyphenols, peculiarly occurring based on the plant organ. At the same time, the possibility of avoiding NaDES removal from the end products without affecting cell health status made them ready-to-use formulations. The joint approach of UHPLC-HRMS techniques to in vitro simulated digestion gave a picture of individual compounds’ bioaccessibility, whose correlation to antiradical assays highlighted that the capitula-based sample retained their efficacy also after gastric and intestinal phases, likely due to the release of apigenin and luteolin glycosides beyond 1,3-dicaffeoylquinic acid. Future perspectives will involve cell metabolomic approaches aimed at deeply investigating cellular uptake and eventual biotransformations as mechanistic effects on polyphenols bioavailability. Moreover, cell-based antioxidant and anti-inflammatory activities will undergo more research to yield further insights into their functionality.

## Figures and Tables

**Table 1:** Putative identification of compounds in NaDES-artichoke extracts, based on UHPLC-HRMS and MS/MS analysis (Rt, retention time; RDB, ring and double bond value; C, capitula; S, stems; L, leaves). Base peak fragment ions are in bold.

Peak n.	Rt (min)	Tentative Assignment	Formula	[M-H]^−^ Calc. (*m*/*z*)	[M-H]^−^ Found (*m*/*z*)	Error (ppm)	RDB	MS/MS Fragment Ions (*m*/*z*)	C	S	L
1	4.606	1-*O*-caffeoylquinic acid	C_16_H_18_O_9_	353.0878	353.0881	0.8	8	353.0863; **191.0563**	×	×	×
2	5.182	5-*O*-caffeoylquinic acid	C_16_H_18_O_9_	353.0878	353.0879	0.3	8	353.0868; **191.0559**	×	×	×
3	5.830	*p*-coumaroylquinic acid 1	C_16_H_18_O_8_	337.0929	337.0929	0	8	337.0921; **191.0560**;173.0441; 119.0508; 93.0343	×	×	×
4	6.174	*p*-coumaroylquinic acid 2	C_16_H_18_O_8_	337.0929	337.0929	0	8	337.0932; **191.0560**; 119.0517	×		×
5	6.227	1,3-dicaffeoylquinic acid (cynarin)	C_25_H_24_O_12_	515.1195	515.1211	3.1	14	515.1209; 353.0878; 335.0796; **191.0553**; 179.0338; 135.0442; 93.0356	×	×	×
6	7.112	Caffeoyl methylquinate 1	C_17_H_20_O_9_	367.1035	367.1042	2.0	8	**367.1050**; 179.0358; 161.0244; 135.0457; 133.0300	×	×	×
7	7.124	Apigenin hexosyl-hexuronide	C_27_H_28_O_16_	607.1305	607.1310	0.9	14	**607.1330**; 431.1007; 269.0449; 268.0378; 175.0252; 113.0238	×		×
8	7.177	Luteolin hexosyl-hexuronide	C_27_H_28_O_17_	623.1254	623.1269	2.4	14	623.1290; 461.0737; 447.0939; **285.0400**; 284.0310;	×	×	×
9	7.300	Eriodictyol hexoside	C_21_H_22_O_11_	449.1089	449.1087	−0.5	11	449.1090; **287.0554**; 193.0124; 151.0026; 107.0127	×	×	×
10	7.308	Caffeoyl methylquinate 2	C_17_H_20_O_9_	367.1035	367.1031	−1.0	8	**367.1032**; 179.0350; 161.0245; 135.0450	×	×	×
11	7.322	Eriodictyol hexuronide	C_21_H_20_O_12_	463.0882	463.0877	−1.1	12	**463.0882**; 287.0556; 151.0041	×		
12	7.449	Eriodictyol rutinoside	C_27_H_32_O_15_	595.1668	595.1676	1.3	12	595.1669; **287.0551**; 151.0033; 135.0440	×		
13	7.500	Quercetin hexuronide	C_21_H_18_O_13_	477.0675	477.0683	1.8	13	477.0678; **301.0341**	×		
14	7.595	Quercetin hexoside	C_17_H_20_O_12_	463.0882	463.0901	4.1	12	**463.0899**; 301.0346; 300.0275	×		
15	7.921	Methyl-luteolin derivative	C_27_H_31_NO_14_	592.1672	592.1685	2.2	13	592.1713; 546.1614; **475.0880**; 299.0542; 285.0400; 284.0308	×		×
16	8.197	Luteolin pentosyl-hexoside	C_26_H_28_O_15_	579.1355	579.1366	1.8	13	**579.1375**; 285.0401	×		
17	8.300	Luteolin hexuronide	C_21_H_18_O_12_	461.0725	461.0733	1.6	13	461.0745; **285.0401**	×	×	×
18	8.397	Luteolin hexoside 1	C_21_H_20_O_11_	447.0933	447.0935	0.5	12	**447.0945**; 285.0396; 284.0322	×	×	×
19	8.493	Luteolin rutinoside (e.g., scolymoside)	C_27_H_30_O_15_	593.1512	593.1526	2.4	13	**593.1551**; 285.0405	×	×	×
20	8.654	1,4-dicaffeoylquinic acid	C_25_H_24_O_12_	515.1195	515.1208	2.5	14	**515.1218**; 353.0884; 335.0772; 191.0557; 179.0352; 173.0447; 135.0451; 93.0337	×	×	×
21	9.045	3,5-dicaffeoylquinic acid	C_25_H_24_O_12_	515.1195	515.1203	1.6	14	515.1195; **353.0868**; 191.0560; 179.0347; 135.0451	×	×	×
22	9.270	1,5-dicaffeoylquinic acid	C_25_H_24_O_12_	515.1195	515.1217	4.3	14	515.1228; 353.0885; **191.0561**	×	×	×
23	9.867	Apigenin hexoside	C_21_H_20_O_10_	431.0984	431.0997	3.1	12	**431.0983**; 269.0438; 268.0361	×	×	×
24	9.869	Apigenin hexuronide	C_21_H_18_O_11_	445.0776	445.0793	3.7	13	445.0787; **269.0441**; 113.0236	×	×	×
25	9.876	Apigenin rutinoside	C_27_H_30_O_14_	577.1563	577.1583	3.5	13	577.1589; **269.0450**	×	×	×
26	9.942	Dicaffeoylquinic acid isomer	C_25_H_24_O_12_	515.1195	515.1200	1.0	14	515.1213; 353.0877; **191.0560**; 179.0343	×	×	×
27	10.104	Luteolin hexoside 2	C_21_H_20_O_11_	447.0933	447.0941	1.8	12	447.0937; **285.0393**	×	×	
28	10.710	3,4-dicaffeoylquinic acid	C_25_H_24_O_12_	515.1195	515.1217	4.3	14	515.1220; **353.0875**; 191.0557; 179.0343;173.0450; 135.0443	×	×	×
29	11.410	Luteolin hexoside 3 (e.g., cynaroside)	C_21_H_20_O_11_	447.0933	447.0949	3.6	12	447.0941; **285.0396**	×		
30	11.584	Luteolin malonylhexoside	C_24_H_22_O_14_	533.0937	533.0947	1.9	14	533.0958; **489.1054**; 285.0406; 284.0323	×		×
31	13.115	Methyl-luteolin hexuronide	C_22_H_20_O_12_	475.0882	475.0903	4.4	13	**475.0888**; 299.0553; 285.0398; 284.0308	×	×	×
32	13.262	Dicaffeoyl methylquinate 1	C_26_H_26_O_12_	529.1352	529.1361	1.8	14	**529.1371**; 367.1030; 349.0922; 179.0350; 161.0246; 135.0452; 133.0298	×	×	×
33	13.277	4,5-dicaffeoylquinic acid	C_25_H_24_O_12_	515.1195	515.1214	3.7	14	515.1230; **353.0889**; 191.0560; 179.0355; 173.0445; 135.0466	×	×	×
34	13.340	Apigenin malonylhexoside	C_24_H_22_O_13_	517.0988	517.1008	3.9	14	473.1085; **269.0446**; 268.0388	×	×	×
35	13.601	Dicaffeoyl methylquinate 2	C_26_H_26_O_12_	529.1352	529.1354	0.5	14	**529.1380**; 367.1031; 179.0353; 161.0244; 135.0454	×	×	×
36	13.618	Apigenin methylhexuronide	C_22_H_20_O_11_	459.0933	459.0943	2.2	13	**459.0935**; 269.0454; 268.0374	×	×	×
37	14.125	Cynarasaponin A (or H)	C_47_H_74_O_19_	941.4752	941.4766	1.5	11	941.4752 *; 779.4192; 735.4324; 717.4237; 645.4011; **629.3673**; 603.3924; 585.3768; 513.3592; 471.3473; 423.3254	×		
38	14.433	Cynarasaponin J	C_47_H_74_O_18_	925.4802	925.4813	1.1	11	925.4802 *; 763.4288; 719.4374; 701.4267; 629.4043; **613.3771**; 587.3979; 569.3849; 523.3807; 497.3642; 455.3528; 453.3382; 407.3288	×		

* Fragmented at higher collision energy value (100 V).

## Data Availability

Data is contained within the article.

## Supplementary Materials

The following supporting information can be downloaded at: https://www.mdpi.com/article/10.3390/foods11243955/s1, Figure S1: TOF-MS/MS spectra of compounds (**a**) **2** and (**b**) **3** and their fragmentation pathways; Figure S2: TOF-MS/MS of compounds (**a**) **7**, (**b**) **23**, (**c**) **24**, and (**d**) **25** and related putative structures; Figure S3: TOF-MS/MS of eriodictyol glycosides (**9**, **11**, and **12**). Pivotal aglycone fragmentations are also highlighted; Figure S4: Hypothesized fragmentation pathway for compounds **37** (*m*/*z* 941.4752) and **38** (*m*/*z* 925.4802). Ring E is representative of cynarasaponin J and H, respectively. Theoretical *m*/*z* values are reported below each structure; Table S1: Release index (%) of compounds in DES-artichoke extracts, based on UHPLC-HRMS analyses. Co, capitula oral phase; Cg, capitula gastric phase; Ci, capitula intestinal phase; So, stems oral phase; Sg, stems gastric phase; Si, stems intestinal phase; Lo, leaves oral phase; Lg, leaves gastric phase; Li, leaves intestinal phase; n.a., not applicable; n.q., not quantifiable; Table S2: Radical scavenging capacity (RCS %) of ready-to-use artichoke formulations before and after simulated digestion protocol, evaluated by DPPH and ABTS tests. Values are reported as mean ± SD. C, not digested capitula; Co, capitula oral phase; Cg, capitula gastric phase; Ci, capitula intestinal phase; S not digested stems; So, stems oral phase; Sg, stems gastric phase; Si, stems intestinal phase; L, not digested leaves; Lo, leaves oral phase; Lg, leaves gastric phase; Li, leaves intestinal phase. Table S3. TEAC (Trolox® Equivalent Antioxidant Capacity, μM) values of ready-to-use artichoke formulations before and after simulated digestion protocol, evaluated by DPPH and ABTS tests. Values are reported as mean ± SD. C = not digested capitula; Co = capitula oral phase; Cg = capitula gastric phase; Ci = capitula intestinal phase; S = not digested stems; So = stems oral phase; Sg = stems gastric phase; Si = stems intestinal phase; L = not digested leaves; Lo = leaves oral phase; Lg = leaves gastric phase; Li = leaves intestinal phase.
