# Supplementary material for: Ready-to-Use Nutraceutical Formulations from Edible and Waste Organs of Algerian Artichokes
Source: Foods. 2022 Dec 7;11(24):3955. doi: 10.3390/foods11243955 (PMC9777799; doi:10.3390/foods11243955)
Supplement: Supplementary file 1 [file foods-11-03955-s001.zip › foods-2061615-supplementary.pdf]

Supplementary materials

Ready-to-Use Nutraceutical Formulations from Edible and Waste Organs of Algerian Artichokes

Nabila Brahmi-Chendouh [1,†], Simona Piccolella [2,†,*], Claudia Gravina [2], Marika Fiorentino [2], Marialuisa Formato [2], Naoual Kheyar [3] and Severina Pacifico [2]

1 Laboratory of Biomathematics, Biochemistry, Biophysics and Scientometry, Faculty of Natural and Life Sciences, University of Bejaia, Bejaia 06000, Algeria
2 Department of Environmental, Biological and Pharmaceutical Sciences and Technologies, University of Campania 'Luigi Vanvitelli', Via Vivaldi 43, 81100 Caserta, Italy
3 Laboratory of Plant Biotechnology and Ethnobotany, Faculty of Nature and Life Sciences, University of Bejaia, Bejaia 06000, Algeria
* Correspondence: simona.piccolella@unicampania.it
† These authors contributed equally to this work.

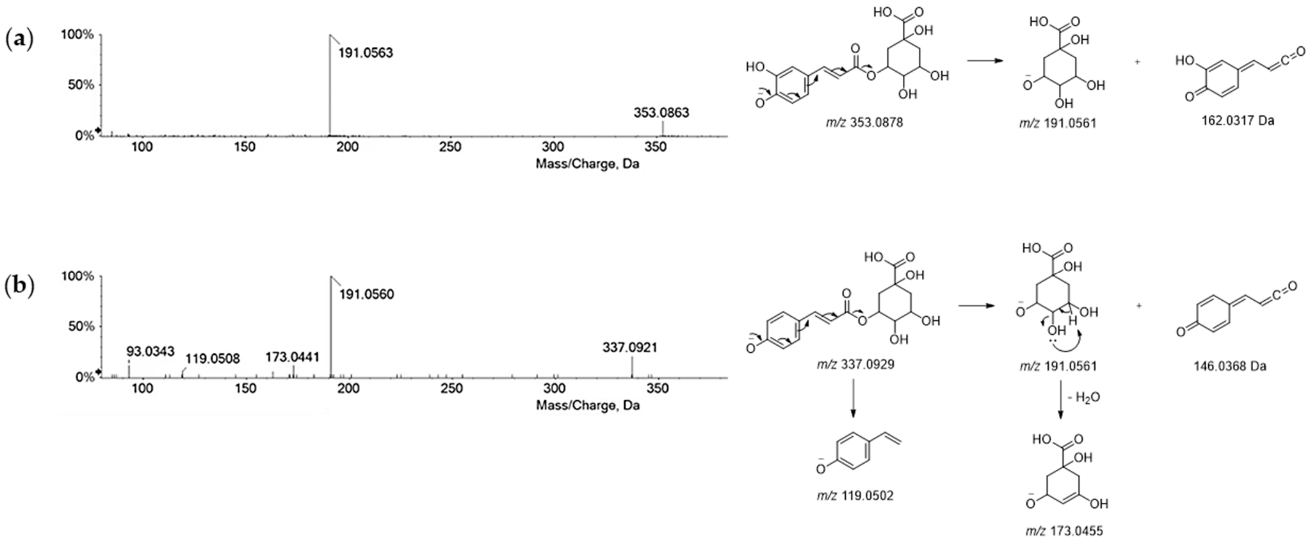

Figure S1. TOF-MS/MS spectra of compounds (**a**) **2**, and (**b**) **3** and their fragmentation pathways.

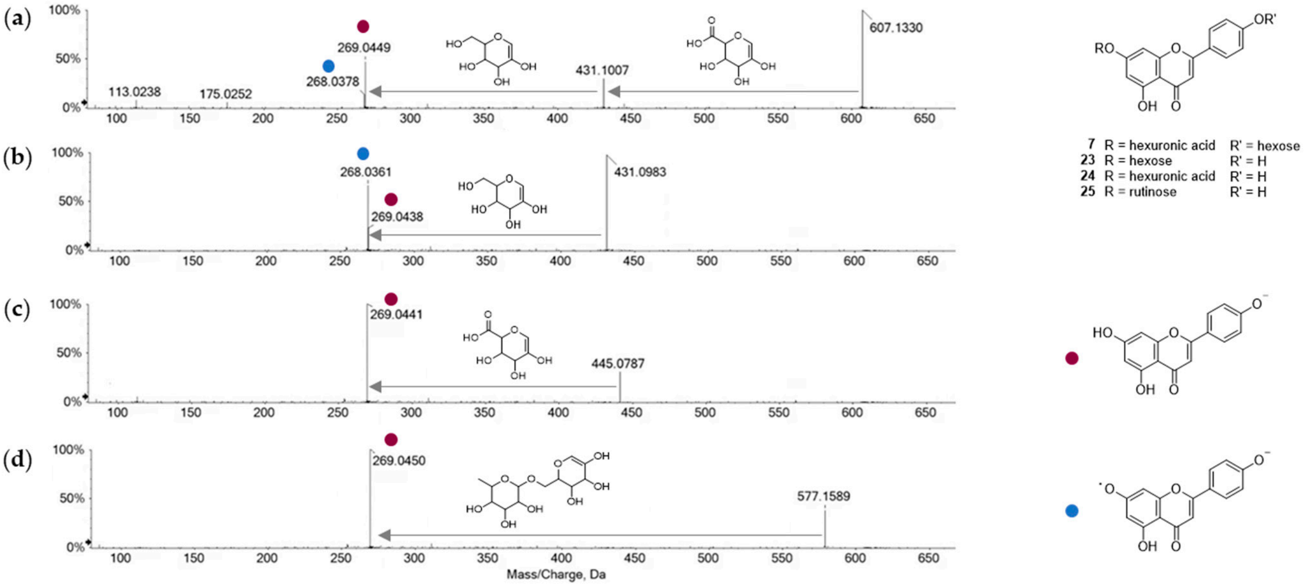

Figure S2. TOF-MS/MS of compounds (**a**) **7**, (**b**) **23**, (**c**) **24**, and (**d**) **25** and related putative structures.

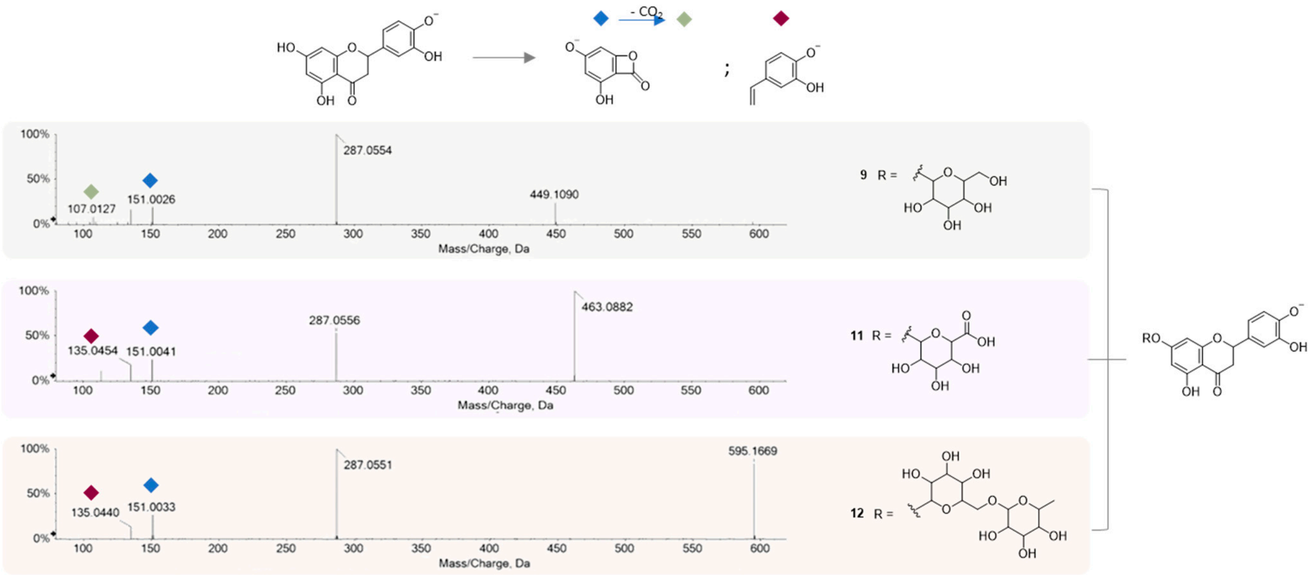

Figure S3. TOF-MS/MS of eriodictyol glycosides (**9**, **11**, and **12**). Pivotal aglycone fragmentations are also highlighted.

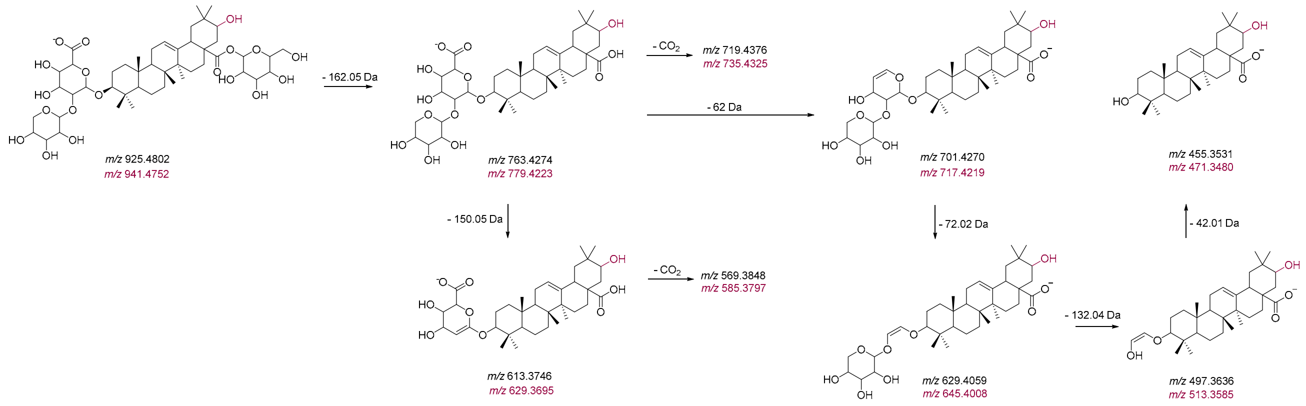

Figure S4. Hypothesized fragmentation pathway for compounds **37** (*m/z* 941.4752) and **38** (*m/z* 925.4802). Ring E is representative of cynarasaponin J and H, respectively. Theoretical *m/z* values are reported below each structure.

Table S1. Release index (%) of compounds in DES-artichoke extracts, based on UHPLC-HRMS analyses. Values are reported as mean ± SD. Co = capitula oral phase; Cg = capitula gastric phase; Ci = capitula intestinal phase; So = stems oral phase; Sg = stems gastric phase; Si = stems intestinal phase; Lo = leaves oral phase; Lg = leaves gastric phase; Li = leaves intestinal phase; n.a. = not applicable; n.q. = not quantifiable.

	**Compound**	**Co**	**Cg**	**Ci**	**So**	**Sg**	**Si**	**Lo**	**Lg**	**Li**
Simple phenols (monoacyl-quinic acid derivatives)										
**1**	1-*O*-caffeoylquinic acid	44.5 ± 0.9	103.9 ± 0.6	44.8 ± 1.2	3.2 ± 0.1	108.1 ± 2.1	2.9 ± 0.0	70.7 ± 1.0	139.1 ± 1.0	22.1 ± 0.0
**2**	5-*O*-caffeoylquinic acid	39.1 ± 1.7	201.0 ± 1.6	37.1 ± 1.5	3.7 ± 0.1	209.7 ± 1.7	3.5 ± 0.0	64.6 ± 1.6	168.5 ± 1.0	13.6 ± 0.3
**3**	*p*-coumaroylquinic acid 1	43.6 ± 0.5	93.9 ± 0.6	46.9 ± 1.6	n.q.	86.0 ± 1.5	15.9 ± 0.3	9.1 ± 0.1	15.6 ± 0.2	7.5 ± 0.3
**4**	*p*-coumaroylquinic acid 2	33.0 ± 1.2	228.7 ± 1.5	79.7 ± 0.7	n.a.	n.a.	n.a.	11.8 ± 0.1	31.2 ± 0.0	9.5 ± 0.3
**6**	Caffeoyl methylquinate 1	74.4 ± 1.1	147.5 ± 0.6	3.8 ± 0.1	3.3 ± 0.0	97.1 ± 0.6	n.q.	36.7 ± 0.5	113.3 ± 0.9	n.q.
**10**	Caffeoyl methylquinate 2	35.5 ± 0.6	90.7 ± 0.9	64.8 ± 0.8	n.q.	101.9 ± 0.9	n.q.	137.1 ± 0.9	182.3 ± 1.2	n.q.
Simple phenols (diacyl-quinic acid derivatives)										
**5**	1,3-dicaffeoylquinic acid (cynarin)	27.9 ± 0.5	80.8 ± 0.5	405.3 ± 1.9	3.0 ± 0.0	264.6 ± 1.2	84.5 ± 1.8	56.7 ± 1.3	409.6 ± 2.0	580.4 ± 2.3
**20**	1,4-dicaffeoylquinic acid	32.7 ± 0.7	216.3 ± 0.9	166.1 ± 1.3	0.7 ± 0.0	191.4 ± 1.0	13.6 ± 0.0	45.6 ± 0.7	133.7 ± 1.4	25.3 ± 0.3
**21**	3,5-dicaffeoylquinic acid	20.5 ± 0.3	90.5 ± 1.2	18.0 ± 0.9	5.8 ± 0.1	138.0 ± 1.1	2.5 ± 0.1	67.0 ± 1.0	107.5 ± 2.5	13.5 ± 0.4
**22**	1,5-dicaffeoylquinic acid	20.9 ± 1.2	80.2 ± 1.0	11.8 ± 1.2	4.0 ± 0.3	98.8 ± 1.1	n.q.	52.8 ± 0.3	111.9 ± 0.4	2.7 ± 0.1
**26**	dicaffeoylquinic acid isomer	14.0 ± 0.8	119.9 ± 1.1	9.2 ± 0.5	2.1 ± 0.1	204.1 ± 0.9	n.q.	45.3 ± 0.7	159.2 ± 0.9	n.q.
**28**	3,4-dicaffeoylquinic acid	21.1 ± 0.2	109.1 ± 0.8	154.7 ± 1.3	14.9 ± 0.9	198.8 ± 1.3	39.8 ± 1.3	104.8 ± 1.0	177.5 ± 0.7	97.3 ± 1.2
**32**	Dicaffeoyl methylquinate 1	53.9 ± 0.4	101.0 ± 1.0	13.1 ± 0.3	3.3 ± 0.1	64.7 ± 0.7	n.q.	33.7 ± 0.5	70.3 ± 0.7	2.0 ± 0.0
**33**	4,5-dicaffeoylquinic acid	21.0 ± 0.3	166.2 ± 1.2	131.8 ± 1.2	3.0 ± 0.1	199.6 ± 1.3	1334.0 ± 3.2	44.5 ± 1.2	184.0 ± 1.1	2199.4 ± 3.5
**35**	Dicaffeoyl methylquinate 2	54.8 ± 0.2	118.1 ± 1.0	52.9 ± 1.0	5.5 ± 0.2	123.2 ± 0.9	n.q.	36.0 ± 0.5	121.4 ± 1.0	5.3 ± 0.1
Polyphenols (apigenin derivatives)										
**7**	Apigenin hexosyl-hexuronide	373.6 ± 1.1	684.6 ± 2.9	29.7 ± 0.2	1.8 ± 0.1	264.7 ± 1.4	n.q.	11.5 ± 0.7	164.8 ± 1.7	n.q.
**23**	Apigenin hexoside	32.9 ± 0.7	108.1 ± 0.9	146.9 ± 1.5	n.q.	122.6	115.2 ± 1.2	145.2 ± 1.2	213.1 ± 2.0	325.2 ± 1.0
**24**	Apigenin hexuronide	21.1 ± 0.9	101.7 ± 1.0	149.0 ± 0.9	n.q.	148.9 ± 0.9	151.3	81.0 ± 1.1	183.7 ± 1.3	220.2 ± 1.3
**25**	Apigenin rutinoside	24.4 ± 1.1	72.6 ± 1.0	128.8 ± 1.5	n.q.	164.7 ± 2.0	92.2 ± 1.2	104.3 ± 0.9	145.8 ± 0.8	240.4 ± 1.2
**34**	Apigenin malonylhexoside	23.2 ± 0.1	93.2 ± 1.4	96.2 ± 1.2	n.q.	n.q.	n.q.	40.3 ± 0.6	142.8 ± 1.4	57.1 ± 0.3
**36**	Apigenin methylhexuronide	57.6 ± 1.2	143.9 ± 0.7	19.9 ± 1.3	4.5 ± 0.3	90.1 ± 1.1	n.q.	18.2 ± 1.3	98.1 ± 1.0	0.7 ± 0.0
Polyphenols (luteolin derivatives)										
**8**	Luteolin hexosyl-hexuronide	29.1 ± 0.4	97.1 ± 0.7	123.3 ± 1.1	n.q.	n.q.	n.q.	126.0 ± 1.2	194.4 ± 0.9	155.9 ± 0.8
**16**	Luteolin pentosyl-hexoside	39.3 ± 0.8	113.5 ± 1.7	151.8 ± 1.5	n.a.	n.a.	n.a.	n.a.	n.a.	n.a.
**17**	Luteolin hexuronide	28.8 ± 0.2	100.2 ± 0.6	114.9 ± 0.9	19.5 ± 0.4	154.5 ± 1.4	100.7 ± 1.1	70.2 ± 0.5	171.7 ± 0.7	150.1 ± 2.2
**18**	Luteolin hexoside 1	41.7 ± 0.8	107.9 ± 1.2	127.0 ± 1.7	11.2 ± 0.6	162.6 ± 1.1	89.2 ± 0.8	112.6 ± 1.2	180.8 ± 0.7	148.1 ± 1.7
**27**	Luteolin hexoside 2	29.8 ± 0.4	141.2 ± 1.6	182.5 ± 1.0	n.q.	n.q.	n.q.	n.a.	n.a.	n.a.
**19**	Luteolin rutinoside (e.g. scolymoside)	40.3 ± 0.6	112.7 ± 0.7	144.4 ± 0.7	8.0 ± 0.5	157.8 ± 2.4	70.7 ± 0.5	64.7 ± 0.8	125.2 ± 1.1	92.0 ± 0.6
**29**	Luteolin hexoside 3 (e.g. cynaroside)	38.9 ± 0.4	147.0 ± 0.6	133.4 ± 1.3	n.a.	n.a.	n.a.	n.a.	n.a.	n.a.
**30**	Luteolin malonylhexoside	24.8 ± 0.7	100.8 ± 0.9	136.5 ± 0.6	n.a.	n.a.	n.a.	n.q.	n.q.	n.q.
**15**	Methyl-luteolin derivative	44.9 ± 1.1	58.5 ± 1.2	n.q.	n.a.	n.a.	n.a.	57.3 ± 0.9	40.0 ± 0.5	27.5 ± 0.3
**31**	Methyl-luteolin hexuronide	40.7 ± 0.5	105.4 ± 1.1	5.2 ± 0.1	3.6 ± 0.1	82.2 ± 0.6	n.q.	36.9 ± 0.4	91.0 ± 1.2	n.q.
Polyphenols (eriodictyol derivatives)										
**9**	Eriodictyol hexoside	26.2 ± 0.8	89.7 ± 1.1	98.9 ± 0.9	n.q.	125.1 ± 0.7	59.8 ± 0.5	114.0 ± 1.2	190.5 ± 1.0	164.2 ± 0.9
**11**	Eriodictyol hexuronide	30.0 ± 0.3	86.3 ± 0.7	66.8 ± 1.2	n.a.	n.a.	n.a.	n.a.	n.a.	n.a.

**12**	Eriodictyol rutinoside	50.9 ± 0.5	119.5 ± 1.0	98.0 ± 1.1	n.a.	n.a.	n.a.	n.a.	n.a.	n.a.
Polyphenols (quercetin derivatives)										
**13**	Quercetin hexuronide	28.7 ± 0.4	91.0 ± 0.8	1.9 ± 0.1	n.a.	n.a.	n.a.	n.a.	n.a.	n.a.
**14**	Quercetin hexoside	39.3 ± 0.5	102.9 ± 2.2	3.6 ± 0.0	n.a.	n.a.	n.a.	n.a.	n.a.	n.a.
Triterpenes										
**37**	Cynarasaponin A (or H)	32.3 ± 0.7	116.5 ± 1.1	37.7 ± 0.4	n.a.	n.a.	n.a.	n.a.	n.a.	n.a.
**38**	Cynarasaponin J	28.5 ± 0.8	115.5 ± 2.1	11.4 ± 0.2	n.a.	n.a.	n.a.	n.a.	n.a.	n.a.

Table S2. Radical scavenging capacity (RCS %) of ready-to-use artichoke formulations before and after simulated digestion protocol, evaluated by DPPH and ABTS tests. Values are reported as mean ± SD. C = not digested capitula; Co = capitula oral phase; Cg = capitula gastric phase; Ci = capitula intestinal phase; S = not digested stems; So = stems oral phase; Sg = stems gastric phase; Si = stems intestinal phase; L = not digested leaves; Lo = leaves oral phase; Lg = leaves gastric phase; Li = leaves intestinal phase.

	Tested dose (μg/mL)	DPPH RSC (%)	$ABTS^+$ RSC (%)		Tested dose (μg /mL)	DPPH RSC (%)	$ABTS^+$ RSC (%)		Tested dose (μg /mL)	DPPH RSC (%)	$ABTS^+$ RSC (%)
**NaDES**	5	-23.0 ± 1.9	-5.0 ± 0.7								
	10	-19.3 ± 1.5	-3.2 ± 0.1								
	50	-15.6 ± 0.6	-2.7 ± 0.2								
	100	-1.8 ± 0.2	-1.4 ± 0.3								
	200	6.9 ± 0.0	-0.6 ± 0.4								
**C**	5	4.0 ± 0.4	2.6 ± 0.5	**L**	5	1.6 ± 0.1	1.5 ± 0.9	**S**	5	1.2 ± 0.2	1.7 ± 1.1
	10	9.1 ± 1.3	12.3 ± 0.3		10	2.2 ± 0.1	3.4 ± 0.5		10	4.0 ± 0.5	3.8 ± 0.6
	50	15.9 ± 0.3	22.8 ± 0.4		50	5.0 ± 0.2	10.1 ± 0.0		50	6.7 ± 0.2	6.6 ± 1.3
	100	43.8 ± 1.3	56.9 ± 0.3		100	30.0 ± 0.5	47.9 ± 1.6		100	16.2 ± 0.2	20.5 ± 0.6
	200	73.9 ± 1.4	62.4 ± 1.4		200	49.1 ± 0.8	57.7 ± 0.3		200	26.5 ± 1.9	29.2 ± 1.4
**Co**	5	-7.7 ± 0.4	-1.7 ± 0.3	**Lo**	5	2.8 ± 0.1	0.8 ± 0.4	**So**	5	-5.4 ± 1.0	8.7 ± 0.6
	10	-3.6 ± 0.2	2.3 ± 0.0		10	6.4 ± 0.1	2.9 ± 0.4		10	-1.1 ± 0.2	14.4 ± 0.6
	50	-2.2 ± 0.4	6.2 ± 0.0		50	8.5 ± 0.7	8.4 ± 0.4		50	7.6 ± 0.1	20.7 ± 0.9
	100	8.3 ± 0.3	26.7 ± 1.2		100	11.0 ± 0.0	13.8 ± 0.3		100	17.0 ± 0.1	32.9 ± 1.7
	200	15.2 ± 1.4	43.5 ± 0.5		200	16.0 ± 0.7	21.4 ± 0.7		200	28.1 ± 0.6	55.2 ± 2.1
**Cg**	5	-9.6 ± 0.3	2.0 ± 0.1	**Lg**	5	-5.4 ± 1.0	6.8 ± 1.4	**Sg**	5	-4.6 ± 0.3	14.2 ± 0.0
	10	-3.8 ± 0.5	10.7 ± 0.8		10	-3.9 ± 2.2	14.9 ± 0.7		10	-0.1 ± 0.1	22.4 ± 0.3
	50	1.6 ± 0.3	18.4 ± 1.2		50	-0.5 ± 0.3	19.7 ± 0.7		50	3.7 ± 0.1	38.9 ± 0.9
	100	8.1 ± 0.6	36.5 ± 0.1		100	4.2 ± 0.0	27.0 ± 0.4		100	6.8 ± 0.7	63.3 ± 0.9
	200	25.0 ± 0.8	56.0 ± 0.2		200	8.9 ± 0.3	34.9 ± 1.8		200	19.4 ± 0.9	86.8 ± 1.0
**Ci**	5	-8.6 ± 0.5	0.7 ± 0.1	**Li**	5	8.3 ± 0.2	8.5 ± 0.7	**Si**	5	-6.3 ± 0.0	1.0 ± 0.3
	10	1.5 ± 0.2	6.9 ± 0.9		10	12.7 ± 0.7	17.2 ± 0.2		10	1.1 ± 0.1	3.6 ± 0.3
	50	7.8 ± 0.7	25.4 ± 1.2		50	13.8 ± 0.3	25.0 ± 0.5		50	2.0 ± 0.2	13.1 ± 0.5
	100	29.0 ± 1.3	52.5 ± 0.1		100	15.1 ± 0.7	42.5 ± 0.4		100	9.3 ± 0.3	35.8 ± 0.7
	200	53.9 ± 1.4	79.7 ± 0.4		200	18.5 ± 1.0	53.1 ± 0.1		200	23.8 ± 0.2	66.1 ± 0.4

Table S3. TEAC (Trolox® Equivalent Antioxidant Capacity, μM) values of ready-to-use artichoke formulations before and after simulated digestion protocol, evaluated by DPPH and ABTS tests. Values are reported as mean ± SD. C = not digested capitula; Co = capitula oral phase; Cg = capitula gastric phase; Ci = capitula intestinal phase; S = not digested stems; So = stems oral phase; Sg = stems gastric phase; Si = stems intestinal phase; L = not digested leaves; Lo = leaves oral phase; Lg = leaves gastric phase; Li = leaves intestinal phase.

	**TEAC DPPH (μM)**	**TEAC ABTS (μM)**		**TEAC DPPH (μM)**	**TEAC ABTS (μM)**		**TEAC DPPH (μM)**	**TEAC ABTS (μM)**
**C**	34.2 ± 0.3	23.4 ± 0.4	**L**	22.6 ± 0.0	21.7 ± 0.2	**S**	12.0 ± 0.5	11.5 ± 0.3
**Co**	6.7 ± 0.3	16.6 ± 0.0	**Lo**	7.1 ± 0.1	8.7 ± 0.0	**So**	8.7± 0.0	32.1 ± 0.1
**Cg**	11.3 ± 0.0	21.1 ± 0.1	**Lg**	3.8 ± 0.3	13.5 ± 0.4	**Sg**	12.8 ± 0.1	20.8 ± 0.6
**Ci**	24.9 ± 0.3	29.6 ± 0.2	**Li**	8.3 ± 0.1	20.1 ± 0.4	**Si**	10.8 ± 0.3	24.7 ± 0.2
